# Comparative effectiveness of recombinant human follicle-stimulating hormone alfa (r-hFSH-alfa) versus highly purified urinary human menopausal gonadotropin (hMG HP) in assisted reproductive technology (ART) treatments: a non-interventional study in Germany

**DOI:** 10.1186/s12958-021-00768-3

**Published:** 2021-06-16

**Authors:** Klaus F. Bühler, Robert Fischer, Patrice Verpillat, Arthur Allignol, Sandra Guedes, Emmanuelle Boutmy, Wilma Bilger, Emilia Richter, Thomas D’Hooghe

**Affiliations:** 1 Department of Gynaecology, Jena-University Hospital-Friedrich Schiller University, 07737 Jena, Germany; 2 Scientific-Clinical Centre for Endometriosis of the University Hospitals of Saarland, 66121 Saarbrücken, Germany; 3 Gynecological Endocrinology and Reproductive Medicine, Fertility Centre Hamburg, 20095 Hamburg, Germany; 4 Global Epidemiology, Research and Development, Merck KGaA, Frankfurter Strasse 250, 64293 Darmstadt, Germany; 5 Medical Affairs Fertility, Endocrinology and General Medicine, Merck Serono GmbH, an affiliate of Merck KGaA, Darmstadt, Germany, Alsfelder Str. 17, 64289 Darmstadt, Germany; 6 Global Medical Affairs Fertility, Research and Development, Merck KGaA, Frankfurter Strasse 250, 64293 Darmstadt, Germany; 7 Department of Development and Regeneration, Laboratory of Endometrium, Endometriosis & Reproductive Medicine, KU Leuven (University of Leuven), Oude Markt 13, 3000 Leuven, Belgium; 8 Department of Obstetrics, Gynecology, and Reproductive Sciences, Yale University Medical School, 333 Cedar St, New Haven, CT 06510 USA

**Keywords:** Real-world data, Recombinant human follicle-stimulating hormone (r-hFSH), Human menopausal gonadotropin (hMG), Follitropin alfa, GONAL-f, Menogon HP

## Abstract

**Background:**

This study compared the effectiveness of recombinant human follicle-stimulating hormone alfa (r-hFSH-alfa; GONAL-f^®^) with urinary highly purified human menopausal gonadotropin (hMG HP; Menogon HP^®^), during assisted reproductive technology (ART) treatments in Germany.

**Methods:**

Data were collected from 71 German fertility centres between 01 January 2007 and 31 December 2012**,** for women undergoing a first stimulation cycle of ART treatment with r-hFSH-alfa or hMG HP. Primary outcomes were live birth, ongoing pregnancy and clinical pregnancy, based on cumulative data (fresh and frozen-thawed embryo transfers), analysed per patient (pP), per complete cycle (pCC) and per first complete cycle (pFC). Secondary outcomes were pregnancy loss (analysed per clinical pregnancy), cancelled cycles (analysed pCC), total drug usage per oocyte retrieved and time-to-live birth (TTLB; per calendar week and per cycle).

**Results:**

Twenty-eight thousand six hundred forty-one women initiated a first treatment cycle (r-hFSH-alfa: 17,725 [61.9%]; hMG HP: 10,916 [38.1%]). After adjustment for confounding variables, treatment with r-hFSH-alfa versus hMG HP was associated with a significantly higher probability of live birth (hazard ratio [HR]-pP [95% confidence interval (CI)]: 1.10 [1.04, 1.16]; HR-pCC [95% CI]: 1.13 [1.08, 1.19]; relative risk [RR]-pFC [95% CI]: 1.09 [1.05, 1.15], ongoing pregnancy (HR-pP [95% CI]: 1.10 [1.04, 1.16]; HR-pCC [95% CI]: 1.13 [1.08, 1.19]; RR-pFC [95% CI]: 1.10 [1.05, 1.15]) and clinical pregnancy (HR-pP [95% CI]: 1.10 [1.05, 1.14]; HR-pCC [95% CI]: 1.14 [1.10, 1.19]; RR-pFC [95% CI]: 1.10 [1.06, 1.14]). Women treated with r-hFSH-alfa versus hMG HP had no statistically significant difference in pregnancy loss (HR [95% CI]: 1.07 [0.98, 1.17], were less likely to have a cycle cancellation (HR [95% CI]: 0.91 [0.84, 0.99]) and had no statistically significant difference in TTLB when measured in weeks (HR [95% CI]: 1.02 [0.97, 1.07]; *p* = 0.548); however, r-hFSH-alfa was associated with a significantly shorter TTLB when measured in cycles versus hMG HP (HR [95% CI]: 1.07 [1.02, 1.13]; *p* = 0.003). There was an average of 47% less drug used per oocyte retrieved with r-hFSH-alfa versus hMG HP.

**Conclusions:**

This large (> 28,000 women), real-world study demonstrated significantly higher rates of cumulative live birth, cumulative ongoing pregnancy and cumulative clinical pregnancy with r-hFSH-alfa versus hMG HP.

**Supplementary Information:**

The online version contains supplementary material available at 10.1186/s12958-021-00768-3.

## Introduction

It is important that assisted reproductive technology (ART) treatment is individualised according to patient characteristics to achieve optimal outcomes [1–4]. This includes the selection of a gonadotropin for use during ovarian stimulation (OS) for ART treatment [5], which is usually based on evaluation of the overall benefits (including effectiveness) and risks of the gonadotropin for each individual patient, in addition to cost effectiveness and patient preferences. Currently available gonadotropins for OS include recombinant human follicle-stimulating hormone (r-hFSH) and urinary human menopausal gonadotropin (hMG), including urinary highly purified hMG (hMG HP). r-hFSH is produced by recombinant DNA technology and only contains FSH activity [6–8]. Follitropin alfa (r-hFSH-alfa, GONAL-f^®^, Merck, KGaA, Darmstadt, Germany), hereafter referred to as r-hFSH-alfa throughout, has a purity of > 99% [9]. In contrast, hMG HP, which is extracted from the urine of postmenopausal women, contains both FSH and luteinizing hormone (LH) activity, as well as other trace proteins [6, 7]. Approximately 95% of the in vivo LH-receptor-mediated bioactivity of hMG HP is attributable to human chorionic gonadotropin [10]. The hMG HP, Menogon HP^®^ (Menopur^®^ [Ferring Pharmaceuticals, Saint-Prex, Switzerland] in Canada, Europe [excluding Germany], South Korea and the USA) is reported to have a purity of ~ 70% [9].

Reflecting differences in manufacturing methods, the FSH content of r-hFSH differs from that of hMG HP in terms of glycosylation pattern (including sialylation) and isoelectric coefficient [6, 7]. The glycosylation pattern of r-hFSH is similar to that observed at the mid-point of the menstrual cycle, whereas hMG HP has a glycosylation pattern seen in menopausal women [6, 7]. Both r-hFSH and hMG HP have an isoelectric profile within the pituitary FSH range [11] and each has a very distinct type of glycosylation [12]. These distinctions could potentially infer differences in efficacy outcomes between r-hFSH and hMG HP. To date, randomised controlled trials (RCTs) comparing these treatments have reported conflicting results, with some RCTs and meta-analyses finding no difference between r-hFSH and urinary gonadotropins (hMG, purified FSH [P-FSH] and highly purified FSH [HP-FSH) [13–15], and others reporting a difference in live birth rate (LBR) and clinical pregnancy rate (CPR) between r-hFSH and hMG [16–19]. The most recent meta-analysis, conducted by Bordewijk et al. in 2019, identified 28 RCTs comparing r-hFSH with urinary-gonadotropins in 7553 women, but only seven of these trials (3397 women) compared r-hFSH with hMG HP [20]. There was no significant difference between the groups in cumulative live birth (three RCTs; 2109 women; relative risk [RR; 95% confidence interval (CI)] 0.91 [0.80, 1.04]). However, considering the aforementioned differences in FSH content and glycosylation patterns as a result of the different manufacturing methods for FSH preparations, since this analysis did not compare one specific r-hFSH product with one specific hMG HP product [20], it does not enable direct comparisons between specific gonadotropins used for OS during ART treatments.

The European Society of Human Reproduction and Embryology (ESHRE) 2019 guidelines equally recommend the use of r-hFSH or hMG for OS [1], based on evidence from a number of RCTs [21–24]; two of which [21, 22] were missing from the Bordewijk meta-analysis [20]. These included an RCT conducted in 749 women reporting a similar cumulative LBR with r-hFSH versus hMG HP (38 vs 40%, respectively) in gonadotropin-releasing hormone (GnRH) antagonist cycles [23], and three RCTs comparing r-hFSH with menotropins (hMG or hMG HP) that reported no significant differences in LBR [21, 22, 24]. These RCTs and the most recent meta-analysis [20] demonstrate the large amount of data available from clinical trials comparing r-hFSH with menotropins. However, these data are from RCTs with strict inclusion/exclusion criteria, usually including a good prognosis population of women younger than 40 years, with regular menstrual cycles, a body mass index (BMI) below 30 and normal ovarian reserve, excluding poor responders [21, 23–29]. This normal responder population typically included in good-quality gonadotropin registration RCTs is reported to reflect only 38% of patients actually treated in a real-world setting [30], and therefore outcomes may differ when evaluated in a real-world population reflective of clinical practice [30–32]. To better reflect clinical practice, real-world data can provide clinicians with additional and valuable information about the long-term effectiveness of a medication in large, heterogeneous populations, thus supplementing data from RCTs and providing reassurance regarding the clinical use of a given treatment [33]. Accordingly, an EU health panel has recently recommended that real-world data should complement RCT data [34].

There have been very few real-world studies of r-hFSH versus hMG HP. One study of 5902 women who underwent 9631 oocyte retrievals and 8818 embryo transfers at two in vitro fertilisation (IVF) centres in Sweden compared LBR between women treated with r-hFSH (follitropin alfa [GONAL-f^®^] and follitropin beta [Puregon^®^]) and those treated with hMG HP (Menopur^®^) [35]. They concluded that LBRs were similar between different treatment groups with both types of gonadotropin when results were adjusted for age and other confounding factors, both in the overall population and in various subgroup analyses. Furthermore, a retrospective chart review of data for 30,630 women in Europe (Germany, Spain, Denmark and Switzerland; the majority from Germany) comparing outcomes in women who received r-hFSH (74%) or hMG HP (26%), observed that a lower mean total gonadotropin dose was used per IVF cycle, and a greater mean number of oocytes retrieved with r-hFSH compared with hMG HP [36]. Although both groups were comparable with respect to the occurrence of a positive pregnancy test and spontaneous abortion, it was not possible to assess the clinical impact of the higher number of oocytes in the r-hFSH arm, as cumulative LBR was not reported [36]. None of these studies compared one specific r-hFSH product with one specific hMG HP product, which is a relevant comparison as biochemical differences between two specific products may result in differences in reproductive outcomes [6, 7].

This study aimed to evaluate the effectiveness of r-hFSH-alfa compared with hMG HP in routine clinical practice in Germany, in terms of cumulative LBR (which is increasingly recognised as the standard clinical approach to measure the success of an ART treatment programme [37–39]), and cumulative ongoing pregnancy and cumulative clinical pregnancy; incorporating both fresh and frozen-thawed embryo transfers [40].

## Materials and methods

### Study design

This was a non-interventional study based on secondary use of data from an electronic database (RecDate) from 71 German IVF centres, which at the time of the study represented 58% of all IVF centres in Germany. RecDate is an established system that was used in reproductive centres by the Deutsches IVF-Register (D∙I∙R) to record and store data for quality assurance purposes; data collected by this system have been previously reported in a number of publications [41–43]. The RecDate system was in place from 1996 until 31 December 2012, after which IVF centres stopped using the RecDate system to report to the D∙I∙R.

All data were anonymised. Data collected in RecDate between 01 January 2007 and 31 December 2012 were analysed. These dates were selected to enable the most recent data within the dataset available to be collected (i.e. most recent at the time of data collection), while allowing adequate follow-up time, since RecDate was no longer used to record and store data for the D∙I∙R after 31 December 2012. The inclusion period for the study was between 01 January 2007 and 31 December 2010. During this period the rate of prospective data in the National Registry (D·I·R) was between 84.0 and 88.0% for all documented cycles and between 92.0 and 81.5% for fresh cycles [44]. Women were included in the analysis until loss to follow-up, treatment switch or the end of the study period (follow-up period ended on 31 December 2012).

### Data collection

The following data were extracted or derived from the database for inclusion in the analysis: baseline variables (including age, BMI, type of infertility, date of last menstrual period, year of first stimulation cycle) and treatment-related variables (hormonal preparation [i.e. type of gonadotropin used]; GnRH protocol [agonist or antagonist]; number of fresh embryos transferred; number of pronuclear-stage embryos [2PN] cryopreserved; ART treatment type [IVF, intracytoplasmic sperm injection (ICSI), IVF + ICSI]; ovarian sensitivity index [OSI], composite variable to measure ovarian response, calculated as: “oocytes recovered x 1000/total dose of FSH” [45]; drug used for final maturation induction; drug used for luteal support and duration of OS).

### Patient population

Women were included in the study if they were undergoing a first stimulation cycle of ART treatment (IVF, ICSI or both) where OS was performed with r-hFSH-alfa, namely follitropin alfa reference product, according to the European Medicines Agency-compliant term to distinguish the preparation reported here from biosimilar preparations [46], or hMG HP between 01 January 2007 and 31 December 2010, and if they used GnRH analogues (either agonist or antagonist) to prevent premature ovulation. The cut-off date of 31 December 2010 was selected in order to analyse adequate follow-up data on pregnancy outcomes over a 2-year time period. Women were excluded from the study if they had co-treatment during a fresh stimulation cycle with clomiphene citrate or a combination of either r-hFSH-alfa or hMG HP with another gonadotropin preparation, or if their first event recorded during the study period was a frozen embryo transfer, as this implied a previous stimulation cycle.

### Definitions and study outcomes

Primary outcomes were measured cumulatively (incorporating both fresh and frozen-thawed embryo transfers) and comprised: cumulative live birth, defined as the number of deliveries that resulted in at least one live birth; cumulative ongoing pregnancy, defined as the number of pregnancies still ongoing at 24 weeks of gestation, with each ongoing multiple pregnancy counted as one ongoing pregnancy; and cumulative clinical pregnancy, defined as the number of pregnancies diagnosed by ultrasonographic visualisation of one or more gestational sacs (multiple gestational sacs are counted as one clinical pregnancy). Data on ovarian hyperstimulation syndrome (OHSS) or ectopic pregnancy were not available for analysis.

Secondary outcome measures were: pregnancy loss (analysed per clinical pregnancy), defined as the number of induced or spontaneous abortions; cancelled cycles (analysed pCC), defined as an ART cycle in which OS or monitoring had been carried out with the intention to treat but no further data were available for this cycle (e.g., did not proceed to follicular aspiration, had no oocytes retrieved or, in the case of a 2PN embryo, did not proceed to embryo transfer); total drug usage per oocyte retrieved (fresh cycle only; analysed descriptively), calculated as the total number of oocytes retrieved per fresh aspiration divided by the total gonadotropin dose; and time-to-live birth (TTLB; analysed per calendar week and per cycle), defined as the time from the date of the first exposure to r-hFSH-alfa or hMG HP to the date of the first pregnancy resulting in a live birth.

A complete ART cycle was defined as all embryos transferred (fresh or frozen) after a single stimulation cycle. Primary outcomes were analysed cumulatively (incorporating both fresh and frozen-thawed embryo transfers), at three levels: 1) cumulatively per patient (pP) (first and all subsequent stimulation cycles and related freeze-thaw cycles for each patient, with each fresh and frozen cycle considered separately in the analysis), 2) cumulatively per complete cycle (pCC) (fresh and frozen transfers for each complete stimulation cycle, with each frozen cycle combined with its respective fresh cycle in the analysis) and 3) cumulatively per first complete cycle (pFC) (first fresh stimulation cycle, fresh embryo transfer and subsequent frozen transfers from the first complete stimulation cycle only) (Supplementary Figure 1).

Primary and secondary outcomes were analysed for the total population and were also stratified according to the GnRH protocol (agonist or antagonist).

### Statistical analysis

pP and pCC analyses were performed using Cox proportional hazards models with a discrete time scale, with unit of time defined as a cycle (hazard ratio [HR] and 95% CI). For example, in a patient undergoing a first fresh cycle followed by a frozen cycle, then a second fresh cycle followed by a frozen cycle (as outlined in Supplementary Figure 1), the pP analysis would contribute four time points, compared with two time points in the pCC analysis. The following outcomes were analysed using Cox proportional hazards models: cumulative live birth, cumulative ongoing pregnancy, cumulative clinical pregnancy, pregnancy loss, cancelled cycles and TTLB analysed per cycle. Analyses of the first stimulation cycle (pFC; comprising cumulative live birth, cumulative ongoing pregnancy and cumulative clinical pregnancy) were performed using a log-binomial regression (RR and 95% CI). Pregnancy loss was analysed per clinical pregnancy; cancelled cycles were analysed pCC. Women were censored if they discontinued treatment or switched to another treatment than the one given for the first cycle; data for these women were only included in the analysis up to the point that treatment was discontinued or switched. Women were also censored if a subsequent stimulation was done without a GnRH analogue. Unadjusted event rates were estimated using the Kaplan-Meier estimator. To control for possible confounding baseline variables, known to be important in the prediction of cumulative live birth [47, 48], we used a propensity score-based approach via inverse probability of treatment weighting [49, 50]. The propensity score offers a versatile tool for transparent confounding adjustment. Inverse probability weighting uses the whole dataset but reweights individuals to increase the weights of those who received unexpected exposures. It generates a pseudo-population with optimal balance of covariates included in the propensity score between treatment groups. The propensity score was estimated using boosted regression trees: a machine learning algorithm that combines many simple decision trees to form a powerful classifier. At each step, women who were incorrectly classified by the previous tree were weighted more heavily than those who were correctly classified. The classifications were then combined to produce the final prediction. A tree depth of three and a learning rate of 0.01 were used. The number of trees was chosen following the method of McCaffrey et al. [49]. This machine learning method has been shown to work better than logistic regression for modelling the propensity score [51].

The relevant covariates to include in the propensity score were chosen by the clinician co-authors (KB, RF, TD) based firstly on factors that have been reported/validated to predict cumulative live birth [47, 48] and secondly on the data that were routinely available in the RecDate database. More background is presented in the Discussion section. The model for propensity scoring included the following baseline confounders: age, BMI, cause of infertility (male factor infertility as reference variable compared with following female causes of infertility reported in the RecDate database: endometriosis, hyperandrogenism/PCOS, endocrine disorders excluding hyperandrogenism/PCOS, tubal pathology, tubal status post sterilisation, uterine or cervical factor infertility, unexplained infertility, psychogenic factor infertility), year of first stimulation cycle initiation, type of GnRH protocol (agonist or antagonist) and ART centre. Due to the potential large weight assigned to extreme observations, a propensity score close to 0 (for the hMG HP) or 1 (for the r-hFSH-alfa) may be problematic for inverse probability of treatment weighting. Therefore, to limit the influence of extreme propensity scores and maximise the clinical equipoise, stabilised weights were used, defined for each patient as:

$$ W=Z\frac{P\left(Z=1\right)}{PS}+\left(1-Z\right)\frac{P\left(Z=0\right)}{1- PS} $$

where Z = 1 if a woman was treated with r-hFSH-alfa, otherwise Z = 0. Covariate balance was assessed before and after weighting by computing standardised mean differences. We considered covariates to be balanced if the absolute value of the standardised mean difference were smaller than 0.1 [52, 53].

In addition to propensity scoring, the following post-treatment variables were included in the final adjusted outcomes models: duration of OS, type of luteal support, type of ART treatment (IVF or ICSI) and the drug used to trigger ovulation. In general, no imputation was performed for missing data, as less than 5% of values were missing for all variables. However, if no data were available on delivery status (which was the case for approximately 2.8% of women), women with an ongoing pregnancy were assumed to have given birth at gestational week 40. Two sensitivity analyses were conducted: the first assessed potential mediation effects due to the inclusion of post-treatment variables in the pregnancy outcome models for the pP analysis, using Cox proportional hazards models that did not include these variables, but which still adjusted for baseline variables using inverse probability weighting. In order to assess the influence of missing live birth information, a second sensitivity analysis was conducted in which all ongoing pregnancy outcomes with missing live birth information were considered as stillbirth.

## Results

### Treatment and baseline patient characteristics

A total of 28,641 women initiated a first treatment cycle with either r-hFSH-alfa or hMG HP: 17,725 (61.9%) women were treated with r-hFSH-alfa and 10,916 (38.1%) were treated with hMG HP. A total of 7296 (25.5%) women initiated a second stimulation cycle with the same gonadotropin as the first cycle, 1783 (6.2%) women initiated a third stimulation cycle and 514 (1.8%) women received > 3 stimulation cycles with the same gonadotropin (fresh cycles).

Baseline characteristics of the unweighted population are shown in Table 1. At baseline, the mean age of women treated with r-hFSH-alfa was lower than the mean age of women treated with hMG HP (33.5 and 35.6 years, respectively). The most frequent infertility diagnoses were ‘male factor’ (57.7 and 51.8% with r-hFSH-alfa and hMG HP, respectively) followed by ‘tubal pathology’ (13.8 and 16.9%, respectively) and ‘idiopathic’ (8.3 and 10.0%, respectively).

**Table 1:** Baseline characteristics of the unweighted population of patients included in the analysis

	r-hFSH-alfa ***N*** = 17,725	hMG HP ***N*** = 10,916	Difference (95% CI)
**Age (years) at index date, mean (SD)**	33.5 (4.4)	35.6 (4.9)	2.07 (1.95, 2.18)
n (%) with non-missing data	17,725 (100)	10,916 (100)	
**BMI (kg/m**^**2**^**) at index date, mean (SD)**	23.6 (4.4)	23.7 (4.4)	0.16 (0.06, 0.27)
n (%) with non-missing data	17,376 (98.0)	10,691 (97.9)	
**Type of infertility, n (%)**			
Male factor	10,046 (57.7)	5545 (51.8)	−5.89 (−7.09, −4.70)
Endometriosis	948 (5.4)	635 (5.9)	0.49 (− 0.07, 1.05)
Hyperandrogenism/PCOS	463 (2.7)	245 (2.3)	−0.37 (− 0.74, 0.00)
Idiopathic	1448 (8.3)	1068 (10.0)	1.66 (0.96, 2.36)
Other	1317 (7.6)	871 (8.1)	0.57 (−0.08, 1.22)
Pathological cycle, other endocrine disorder^a^	650 (3.7)	417 (3.9)	0.16 (−0.30, 0.62)
Psychological factors	7 (0.0)	5 (0.1)	0.01 (−0.04, 0.06)
Status post sterilisation	21 (0.1)	56 (0.5)	0.40 (0.26, 0.55)
Tubal pathology	2410 (13.8)	1804 (16.9)	3.01 (2.14, 3.89)
Uterine, cervical factor	109 (0.6)	63 (0.6)	−0.04 (−0.22, 0.15)
**Year of first stimulation cycle, n (%)**			
2007	4696 (26.5)	2688 (24.6)	−1.87 (− 2.91, −0.83)
2008	4002 (22.6)	2612 (23.9)	1.35 (0.34, 2.36)
2009	4571 (25.8)	2879 (26.4)	0.59 (−0.46, 1.63)
2010	4455 (25.1)	2736 (25.1)	−0.07 (−1.10, 0.96)

*BMI* body mass index, *CI* confidence interval, *PCOS* polycystic ovary syndrome, *SD* standard deviation

^a^Excluding hyperandrogenism and polycystic ovary syndrome

**Fig. 1:**
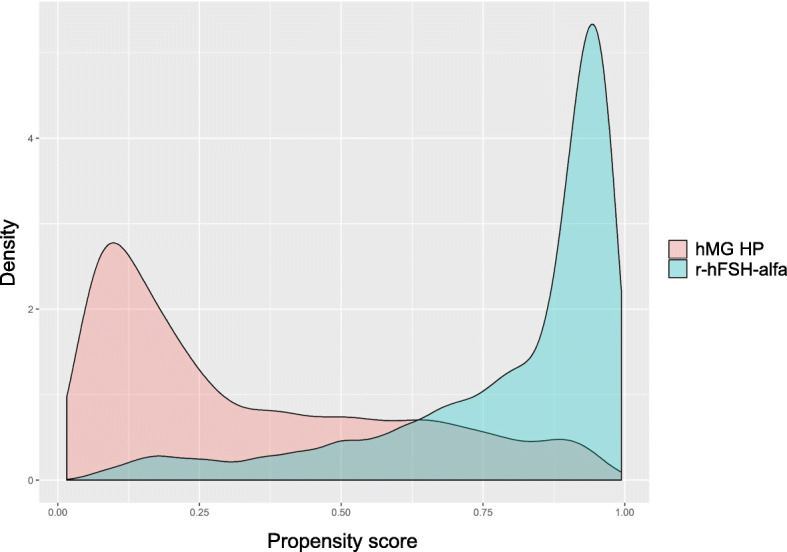
Propensity score distribution between cohorts before weighting. Propensity score distribution between cohorts before weighting. The propensity score is the probability of receiving r-hFSH-alfa. The good overlap between these curves demonstrates that there is enough (empirical) equipoise; i.e. there are enough patients with a probability higher than 0 to receive both treatments to allow for a meaningful comparison

**Fig. 2:**
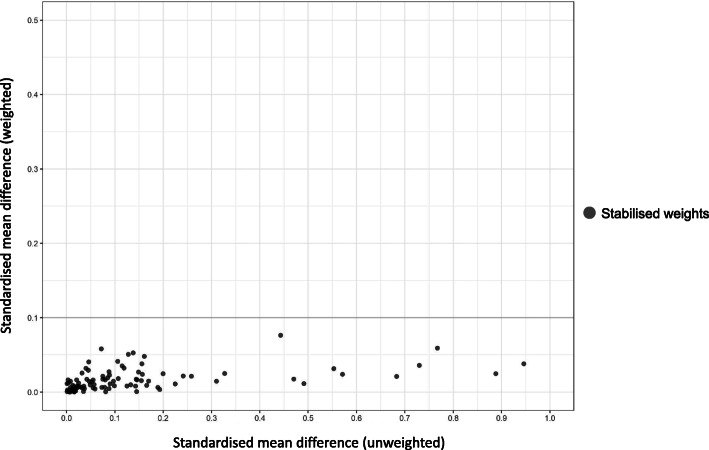
Standardised mean differences before and after weighting. Covariates were considered to be balanced if the absolute value of the standardised mean difference was < 0.1

Treatment characteristics are shown in Table 2. The majority of women in the study used a GnRH agonist (74.4% with r-hFSH-alfa and 81.3% with hMG HP), with the long agonist protocol being the most frequently used (65.9% with r-hFSH-alfa and 53.0% with hMG HP). A GnRH antagonist was used by 25.6% of women receiving r-hFSH-alfa and 18.7% of women receiving hMG HP. For both groups, progesterone was the most frequent luteal support (51.7% with r-hFSH-alfa and 41.7% with hMG HP). The mean [standard deviation (SD)] number of embryos transferred was comparable in the r-hFSH-alfa (1.9 [0.7]) and in the hMG HP (1.8 [0.8]) groups. A greater number of 2PN embryos were cryopreserved in women who received r-hFSH-alfa compared with women who received hMG HP (mean [SD] 2.1 [3.4] vs 1.2 [2.6], respectively). The propensity score showed good overlap between r-hFSH-alfa and hMG HP (see Fig. 1). The distributions of absolute standardised differences between treatment groups before (unweighted) and after propensity score weighting (weighted) are summarised in Fig. 2. After propensity score weighting, all standardised mean differences were < 0.1, demonstrating that the propensity score had been successfully adjusted for all the confounders included in the model. All the results below were adjusted for the variables listed in the statistical analysis section.

**Table 2:** Treatment-related characteristics of the unweighted population, for stimulation cycles (fresh only)

	r-hFSH-alfa ***N*** = 23,429	hMG HP ***N*** = 14,805	Difference (95% CI)
GnRH protocol, n (%)			
Agonist	17,434 (74.4)	12,031 (81.3)	6.85 (6.01, 7.69)
Long	15,438 (65.9)	7845 (53.0)	−12.90 (− 13.91, − 11.90)
Short	1670 (7.1)	4007 (27.1)	19.94 (19.15, 20.73)
Ultralong	279 (1.2)	143 (1.0)	− 0.22 (− 0.43, − 0.01)
Ultrashort	47 (0.2)	36 (0.2)	0.04 (−0.06, 0.14)
Antagonist	5995 (25.6)	2774 (18.7)	−6.85 (−7.69, −6.01)
Multiple	3834 (16.4)	2010 (13.6)	−2.79 (−3.52, −2.06)
Single	2161 (9.2)	764 (5.2)	−4.06 (−4.58, − 3.55)
Mean (SD) number of oocytes retrieved	10.3 (6.2)	8.2 (5.8)	−2.02 (− 2.15, −1.90)
Mean (SD) number of embryos transferred	1.9 (0.7)	1.8 (0.8)	−0.05 (−0.06, −0.03)
Mean (SD) number of 2PN cryopreserved	2.1 (3.4)	1.2 (2.6)	−0.84 (− 0.90, − 0.78)
ART treatment, n (%)			
ICSI	17,013 (72.6)	9471 (64.0)	−8.64 (−9.60, −7.68)
IVF	5436 (23.2)	4605 (31.1)	7.90 (6.98, 8.82)
IVF, ICSI	611 (2.6)	190 (1.3)	−1.32 (−1.60, −1.05)
Not planned	369 (1.6)	539 (3.6)	2.07 (1.72, 2.41)
Mean (SD) OSI (oocytes per 1000 IU)	10.8 (16.0)	7.7 (22.1)	−3.05 (−3.46, −2.64)
Drug used to trigger ovulation, n (%)			
r-hCG	13,164 (57.4)	5579 (39.1)	−18.29 (−19.31, −17.26)
Triptorelin	37 (0.2)	7 (0.1)	−0.11 (−0.18, −0.05)
u-hCG	9694 (42.3)	8612 (60.4)	18.10 (17.07, 19.12)
Other	43 (0.2)	70 (0.5)	0.30 (0.18, 0.43)
Drug used for luteal support, n (%)			
Progestogens	12,113 (51.7)	6167 (41.7)	−10.05 (−11.07, −9.03)
Estrogen	5 (0.0)	9 (0.1)	0.04 (0.00, 0.08)
hCG	217 (0.9)	362 (2.5)	1.52 (1.24, 1.80)
Data not available	2732 (11.7)	2969 (20.1)	8.39 (7.63, 9.16)
Other	26 (0.1)	6 (0.0)	−0.07 (−0.12, −0.02)
Progestogens and estrogen	3367 (14.4)	1675 (11.3)	−3.06 (−3.74, −2.38)
Progestogens and hCG	4421 (18.9)	3150 (21.3)	2.41 (1.58, 3.23)
Progestogens, estrogen and hCG	548 (2.3)	467 (3.2)	0.82 (0.47, 1.16)
Mean (SD) duration of COS, days	10.8 (2.4)	10.8 (2.6)	−0.01 (−0.07, 0.04)
Mean (SD) total drug usage (IU)	1546.3 (875.4)	2147.0 (1330.7)	600.74 (576.55, 624.93)
Mean (SD) total drug usage per oocyte retrieved (IU)	236.0 (332.2)	455.4 (687.0)	219.43 (207.58, 231.29)

*ART* assisted reproductive technology, *CI* confidence interval, *OS* ovarian stimulation, *GnRH* gonadotropin-releasing hormone, *ICSI* intracytoplasmic sperm injection, *IVF* in vitro fertilisation, *OSI* ovarian sensitivity index, *r-hCG* recombinant human chorionic gonadotropin, *SD* standard deviation, *u-hCG* urinary human chorionic gonadotropin

### Primary outcomes

**Fig. 3:**
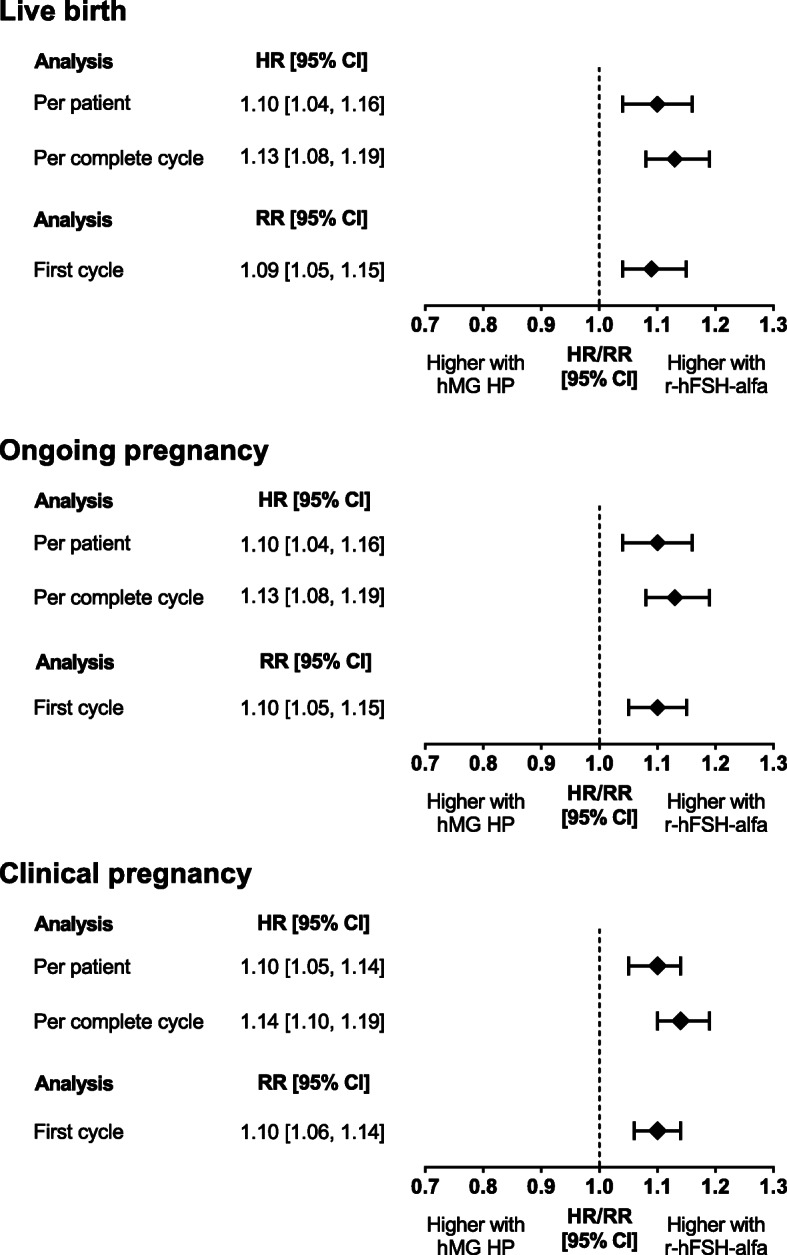
Primary outcomes in the overall population adjusted for possible confounding factors. Differences between study groups were adjusted for possible baseline confounding factors *(age, BMI, type of infertility, GnRH protocol, year of first cycle and IVF centre)* via inverse probability of treatment weighting using a propensity score estimated by boosted regression trees. Data were also adjusted for the following post-treatment variables: duration of OS, type of luteal support, type of ART treatment and the drug used to trigger ovulation. Data were analysed cumulatively (i.e. a complete cycle included all fresh and frozen transfers following a single stimulation cycle). HR, hazard ratio; RR, relative risk

Cumulative LBR was higher in women receiving r-hFSH-alfa compared with hMG HP (HR-pP [95% CI]: 1.10 [1.04, 1.16]; HR-pCC: 1.13 [1.08, 1.19]; RR-pFC: 1.09 [1.05, 1.15]) (Fig. 3). Women treated with r-hFSH-alfa compared with hMG HP had a higher cumulative ongoing pregnancy rate (OPR) (HR-pP [95% CI]: 1.10 [1.04, 1.16]; HR-pCC: 1.13 [1.08, 1.19]; RR-pFC: 1.10 [1.05, 1.15]) and cumulative CPR (HR-pP [95% CI]: 1.10 [1.05, 1.14]; HR-pCC: 1.14 [1.10, 1.19]; RR-pFC: 1.10 [1.06, 1.14]) (Fig. 3).

The results observed in the GnRH agonist-treated sub-population were comparable to the results of the overall population (Supplementary Figure 2). No statistically significant difference in cumulative LBR, CPR and OPR between r-hFSH-alfa and hMG HP were observed in the GnRH antagonist-treated sub-population (Supplementary Figure 2).

### Secondary outcomes

**Fig. 4:**
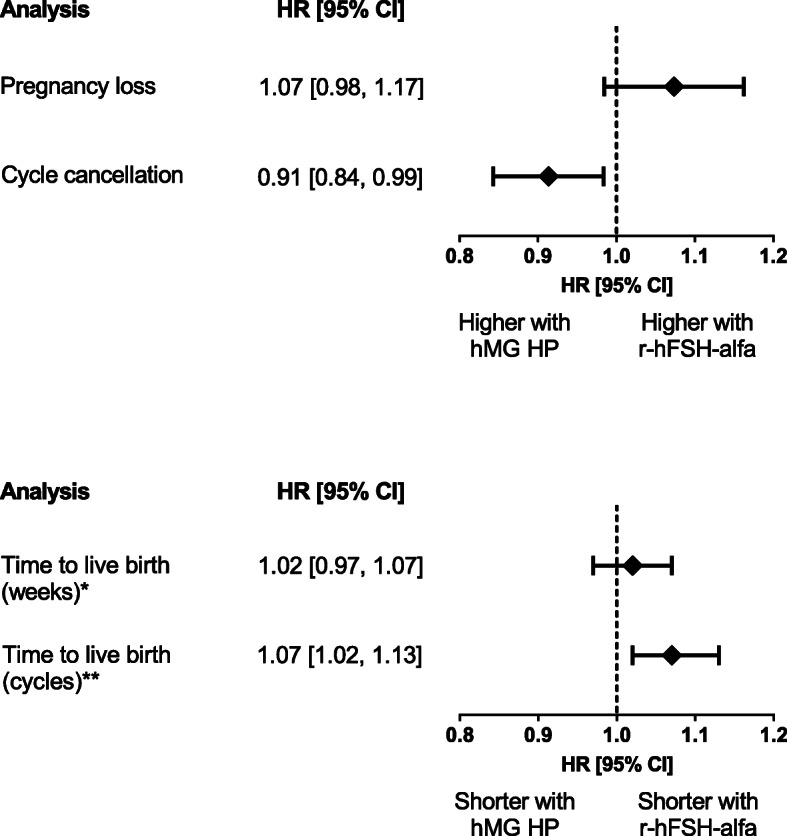
Secondary outcomes in the overall population adjusted for possible confounding factors. Differences between study groups were adjusted for possible confounding factors via inverse probability of treatment weighting using a propensity score estimated by boosted regression trees. HR, hazard ratio. *p = 0.548; **p = 0.003

There was no statistically significant difference in pregnancy loss between women treated with r-hFSH-alfa when compared to women treated with hMG HP (HR [95% CI]: 1.07 [0.98, 1.17]; Fig. 4). Women receiving r-hFSH-alfa were less likely to have a cycle cancellation than women receiving hMG HP (HR [95% CI]: 0.91 [0.84, 0.99]; Fig. 4). There was no statistically significant difference between the two treatments in TTLB when measured in weeks (HR [95% CI]: 1.02 [0.97, 1.07]; *p* = 0.548), but r-hFSH-alfa was associated with a significantly shorter TTLB when measured in cycles compared to hMG HP (HR [95% CI]: 1.07 [1.02, 1.13]; *p* = 0.003; Fig. 4). There was an average of 47% less drug used per oocyte retrieved with r-hFSH-alfa compared with hMG HP (mean [SD]: 236.0 IU [332.2] vs 455.4 IU [687.0], respectively; Table 2). A higher OSI was observed with r-hFSH-alfa compared with hMG HP (median [IQR] 6.7 [3.6–12.5] vs 3.8 [1.9–7.8], respectively).

When secondary outcome analysis was stratified by GnRH protocol, outcomes generally remained similar to those seen in the total population (Supplementary Figure 3). However, while in the overall population r-hFSH-alfa was associated with a significantly shorter TTLB (measured in cycles) compared to hMG HP, in the GnRH antagonist sub-population there was no significant difference between the two groups for this outcome (adjusted HR [95% CI]: 0.97 [0.86, 1.09]). Furthermore, while women in the overall population were less likely to have a cycle cancellation with r-hFSH-alfa, there was no significant difference between groups in this outcome in the GnRH antagonist sub-population (adjusted HR [95% CI]: 0.96 [0.80, 1.14]). However, it is important to note that only a minority of women in the study received a GnRH antagonist protocol (23%).

The results of the two sensitivity analyses were consistent with the main outcomes (data not shown).

## Discussion

This study compared the effectiveness of r-hFSH-alfa and hMG HP, the two most commonly prescribed gonadotropins for ART treatments in Germany (at the time of study), in a large real-world German population. Women treated with r-hFSH-alfa had a significantly higher cumulative LBR, OPR and CPR compared with those treated with hMG HP. In addition, the risk of cycle cancellation and the total drug used per oocyte retrieved were lower in women treated with r-hFSH-alfa rather than hMG HP. TTLB measured in cycles was also shorter with r-hFSH-alfa compared with hMG HP, but this difference was not seen when TTLB was measured in weeks, probably because the measurement in weeks included both the treated and untreated periods, resulting in more variability due to factors such as treatment delays and patient decision making [54]. This study is still relevant today as, although urinary gonadotropins are increasingly being replaced by recombinant gonadotropins in Germany, the D∙I∙R annual report for 2019 stated that 15.3% of stimulated ART cycles included hMG alone or in combination with r-hFSH [55]. Furthermore, a worldwide study reported that 16.4% of clinicians only/mostly prescribed urinary gonadotropins [56]. At the time of the study the preferential use of GnRH agonists was common practice in Germany, which explains why the majority of women in our analysis received a GnRH agonist (77%), with a long agonist protocol most frequently used. As it has previously been observed that the type of GnRH analogue used can affect reproductive outcomes, including pregnancy rates [54, 57–59], we thought it was important to assess outcomes not only in the overall population, but also in the GnRH agonist and GnRH antagonist sub-populations. Although the current study was not designed to directly compare GnRH agonist protocols with GnRH antagonist protocols, differences in outcomes were observed between these two approaches. For example, although outcomes were similar to the overall population in women receiving a GnRH agonist, no significant differences in cumulative LBR, OPR and CPR were observed between r-hFSH-alfa and hMG HP in women treated with a GnRH antagonist, which is in contrast to the overall population. These differences may be related to the fact that only a minority of women in the study followed a GnRH antagonist protocol: fewer cycles used antagonists than used agonists, and the pregnancy rate per embryo transfer was lower in the antagonist cycles than in the agonist cycles. At the time of our study, GnRH antagonists were more likely to be prescribed in older women with previously failed IVF cycles [60–62], who were more likely to have a poor a priori response to OS, which may have also contributed to lower success rates with this protocol. Furthermore, a lack of clinical experience at the time may have resulted in a lower success rate for antagonist protocols [60, 61], since these protocols may not have been used as efficiently as they are today. Accordingly, this lack of experience with antagonist usage and an aversion to prescribe protocols that may have had even a slightly lower success rate may have contributed to the lower usage at the time [60, 61]. By comparison, in the annual reports from the D∙I∙R for the years 2016, 2017 and 2018, antagonist protocols became the major protocol and the pregnancy rates per embryo transfer moved closer to those reported for agonist cycles [63–65], which can be expected as the most recent meta-analyses have shown similar reproductive outcomes after use of GnRH agonist or antagonist [66, 67]. It is important to note that any differences between antagonist and agonist protocols in our study should be taken as descriptive only, due to smaller sample size for the GnRH antagonist protocol.

The difference in outcomes with r-hFSH-alfa and hMG HP observed in our real-world study is not in agreement with some published RCTs and meta-analyses comparing r-hFSH and menotropins, many of which reported conflicting results; some finding no difference in LBR and CPR [13–15, 21–23] and some reporting a difference between treatments in favour of menotropins [16–20, 23]. There may be several reasons for these discrepancies. Firstly, our study directly compared treatments with two specific gonadotropins, each with their specific biochemical properties as outlined in the Introduction section, whereas previous systematic reviews and meta-analyses comprised combinations of different types of r-hFSH and menotropins, potentially masking any treatment differences between specific products. Secondly, differences may result from the data in our study being analysed cumulatively, whereby further treatment cycles were only included in the analysis if they were done with the same initial treatment, with no switch between treatments permitted. However, in this dataset treatment switches did not occur that frequently during the study. Although the number of oocytes and embryos per OS cycle were not compared in this study, it is well known that, for an equal starting dose, a higher number of oocytes and embryos is obtained after OS with r-hFSH-alfa than with hMG HP [23, 25, 68, 69], and that a higher number of available oocytes and embryos can correlate with an increased cumulative LBR [70, 71]. The fact that a greater number of 2PN embryos were cryopreserved in the r-hFSH-alfa arm compared with the hMG HP arm supports this hypothesis. Thirdly, the population investigated in our study represents a real-world patient group derived from a national registry, without the stringent inclusion and exclusion criteria usually required for inclusion in RCTs. Accordingly, the use of real-world data provides us with large sample sizes to assess the comparability of two treatments in all patients treated (e.g., regardless of age or predicted response), whereas only women with normal ovarian reserve (expected normal responders) are typically included in good-quality gonadotropin registration RCTs, which would reflect only 38% of patients actually treated in a real-world setting [30].

A critical question regarding the validity of our results is whether the patient population treated with r-hFSH-alfa and treated with hMG are comparable. We ensured this by including all available baseline factors, known and validated to predict cumulative live birth based on the best available and validated models predicting cumulative LBR [47, 48, 72, 73] as confounding variables in the propensity score method. These baseline factors included age, cause of infertility [47, 48], BMI [48], year of OS for ART [47] and type of centre [72, 74] but we could not include other factors, like duration of infertility [47] or occurrence/outcome of previous pregnancy [47, 48] as these data were not recorded and therefore were not available in the RecData database. In addition to propensity scoring, we included the following post-treatment variables in the final adjusted outcomes models: duration of OS, drug used to trigger ovulation, laboratory method for ART treatment (IVF or ICSI) [47] and type of luteal phase support. We did not include the number of oocytes or embryos retrieved, as these outcomes are affected by the two treatment options compared in this study: a higher number of oocytes and embryos are obtained after OS with r-hFSH-alfa compared to OS with hMG, as outlined in previous paragraph. We also did not include baseline ovarian reserve biomarkers like antral follicle count, serum anti-Müllerian hormone or Day 3 basal FSH, as these variables were not included in the validated prediction models for cumulative LBR [47, 48, 72]. This is in line with evidence from a systematic review/meta-analysis [75] and more recent prospective observational [76] and USA Society for Assisted Reproductive Technology registry [77] studies that these variables, independent of female age, are poor predictors of live birth, and should not be used to alter clinical decisions, even though they can adequately predict low and excessive response to OS for ART [75].

This real-world study has a number of strengths, one of which is the RecDate database itself, which has been well established in providing quality data in the field of reproductive medicine [41–43]. At the time of the study, the RecDate platform was part of the data recording for D∙I∙R, but collected more items in comparison with D∙I∙R. The reliability and quality of the data are strengthened by the fact that RecDate is controlled by an independent IT institution that anonymises the data and corrects/completes data if necessary. Excluding women who switched treatment would have led to immortal-time bias. Therefore, we censored such observations at the time of discontinuation or switch, which helped to increase the thoroughness of the data analysis, and may explain why the results from the primary outcomes were consistent across the different analyses (pP, pCC). Furthermore, as explained in the Methods section, and in the Discussion paragraph above, the propensity score method was a further strength and helped to adjust for known confounders at baseline and provided confidence in the interpretation of the data. The machine learning algorithm with boosted regression trees method to estimate the propensity score has shown good properties to optimise propensity score estimation [49, 51]. Stabilised weights helped maximise the clinical equipoise at baseline and minimise contrasts among comparable treatment groups, as assessed by the standardised mean differences that were all smaller than 0.1 after propensity score weighting.

This study has some limitations that should be acknowledged. Propensity scores directly address the determinants of treatment, driving researchers to think through the clinical decision-making process and the potential sources of confounding of the exposure outcome association [78]. This method can be used to address the lack of randomisation in real-world studies,, minimizing the effect of known confounding variables. Nonetheless, one of the main limitations of the propensity score method is that there is no way of incorporating the effect of potential unknown confounders, and some potentially confounding baseline variables, such as duration of infertility, primary versus secondary infertility, occurrence and outcome of previous pregnancy (if applicable), and the prescribing of medications according to a patient’s ability to pay, were not available for the analysis, although we would not expect these to be a cause of bias, as we observed a substantial overlap between the distribution of propensity scores by treatment groups. Artificially censoring women at their time of discontinuation or switch may lead to issues with the independent censoring assumption needed in time-to-event analysis. This assumption states that women who are censored at a particular time are representative of the women who are still in the study at the same time point. In addition, the data used in the analysis were dependent on the accuracy of the physician recording the information, with the potential for missing outcome parameters or follow-up data, which could have led to possible misclassification. There is, however, no reason this misclassification would be influenced by the treatment prescribed, so would be unlikely to cause a differential bias between the two cohorts. A further limitation was that OHSS and other safety outcomes were not included in the study, since these data were not available in the database.

## Conclusions

The effectiveness of r-hFSH-alfa (GONAL-f^®^) and hMG HP were compared in a large (> 28,000 women), real-world population. Cycles stimulated with r-hFSH-alfa versus hMG HP had increased cumulative LBR, CPR and OPR, alongside decreased cancellation rate and gonadotropin usage per oocyte retrieval in the overall population and in the sub-population of women treated with GnRH agonists. TTLB measured in cycles was also shorter with r-hFSH-alfa versus hMG HP, although no differences in TTLB measured in weeks was observed between the two treatments. The results in women receiving GnRH agonists were similar to the overall results.

## Supplementary Information

**Additional file 1: Supplementary Figure 1.** Study design. **Supplementary Figure 2.** Primary outcomes stratified by GnRH protocol adjusted for possible confounding factors. **Supplementary Figure 3.** Secondary outcomes stratified by GnRH protocol adjusted for possible confounding factors.

## Data Availability

For all new products or new indications approved in both the European Union and the USA after 1 January, 2014, Merck KGaA (Darmstadt, Germany) will share patient- and study-level data after deidentification, as well as redacted study protocols and clinical study reports from clinical trials in patients. These data will be shared with qualified scientific and medical researchers, upon a researcher’s request, as necessary for conducting legitimate research. Such requests must be submitted in writing to the company’s data sharing portal and will be internally reviewed regarding criteria for researcher qualifications and legitimacy of the research purpose.

## Abbreviations

2PN: Pronuclear-stage embryos
ART: Assisted reproductive technology
BMI: Body mass index
CI: Confidence interval
CPR: Clinical pregnancy rate
D∙I∙R: Deutsches IVF-Register
ESHRE: European Society of Human Reproduction and Embryology
GnRH: Gonadotropin-releasing hormone
hMG: Urinary human menopausal gonadotropin
hMG HP: Urinary highly purified hMG
HR: Hazard ratio
ICSI: Intracytoplasmic sperm injection
IVF: In vitro fertilisation
LBR: Live birth rate
LH: Luteinizing hormone
OHSS: Ovarian hyperstimulation syndrome
OPR: Ongoing pregnancy rate
OS: Ovarian stimulation
OSI: Ovarian sensitivity index
pCC: Per complete cycle
pFC: Per first complete cycle
pP: Per patient
RCTs: Randomised controlled trials
r-hFSH: Recombinant human follicle-stimulating hormone
r-hFSH-alfa: Follitropin alfa
RR: Relative risk
SD: Standard deviation
TTLB: Time-to-live birth
