# Supplementary material for: Comparative effectiveness of recombinant human follicle-stimulating hormone alfa (r-hFSH-alfa) versus highly purified urinary human menopausal gonadotropin (hMG HP) in assisted reproductive technology (ART) treatments: a non-interventional study in Germany
Source: Reprod Biol Endocrinol. 2021 Jun 16;19:90. doi: 10.1186/s12958-021-00768-3 (PMC8207759; doi:10.1186/s12958-021-00768-3)

# Supplementary appendix

**Figure legends**

**Supplementary Figure 1.** Study design

*All stimulation cycles and related freeze-thaw cycles for each patient represent a time point in the analysis (contributing four time points in this example), with each fresh and frozen cycle considered separately; ^†^Fresh and frozen transfers for each complete stimulation cycle represent a time point in the analysis (contributing two time points in this example), with each frozen cycle combined with its respective fresh cycle; ^‡^Only the first fresh stimulation cycle, fresh embryo transfer and subsequent frozen transfers from the first complete stimulation cycle were included. A complete ART cycle includes all embryos transferred (fresh or frozen) after a stimulation cycle resulted from the same treatment. The study design is a comparative cohort including patients treated with r-hFSH-alfa (first cohort), and patients treated with hMG HP (second cohort).

**Supplementary Figure 2.** Primary outcomes stratified by GnRH protocol adjusted for possible confounding factors

Differences between study groups were adjusted for possible confounding factors *(age, BMI, type of infertility, GnRH protocol, year of first cycle and IVF centre*) via inverse probability of treatment weighting using a propensity score estimated by boosted regression trees. Data were also adjusted for the following post-treatment variables: *duration of COS, type of luteal support, type of ART treatment, and the drug used to trigger ovulation*. Data were analysed cumulatively (i.e. a complete cycle included all fresh and frozen transfers following a single stimulation cycle). HR, hazard ratio; RR, relative risk

**Supplementary Figure 3.** Secondary outcomes stratified by GnRH protocol adjusted for possible confounding factors

Differences between study groups were adjusted for possible confounding factors via inverse probability of treatment weighting using a propensity score estimated by boosted regression trees. *p=0.044; ^†^p<0.001; ^‡^p=0.016; ^§^p=0.595. HR, hazard ratio

# Supplementary Figure 1


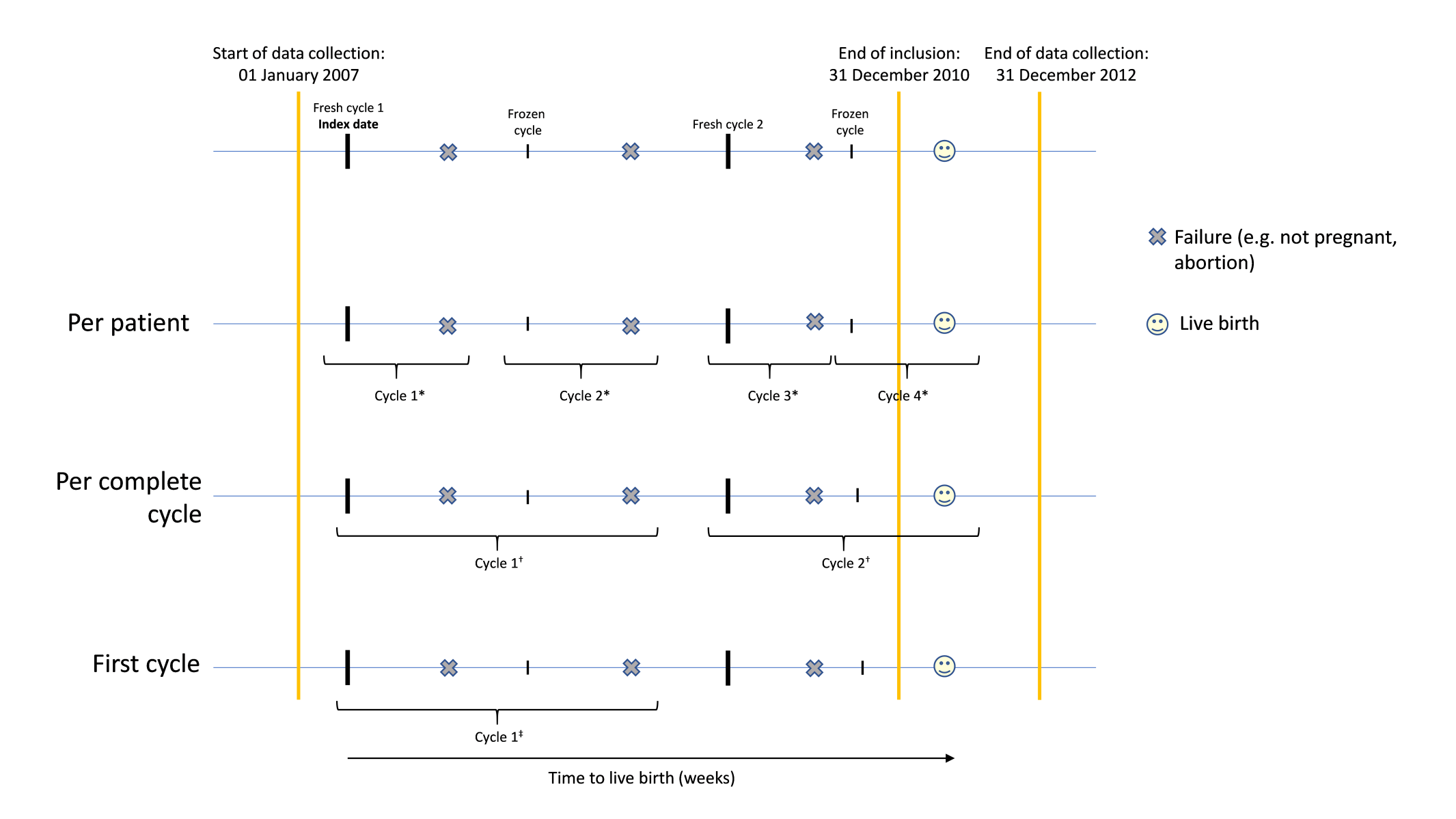


# Supplementary Figure 2


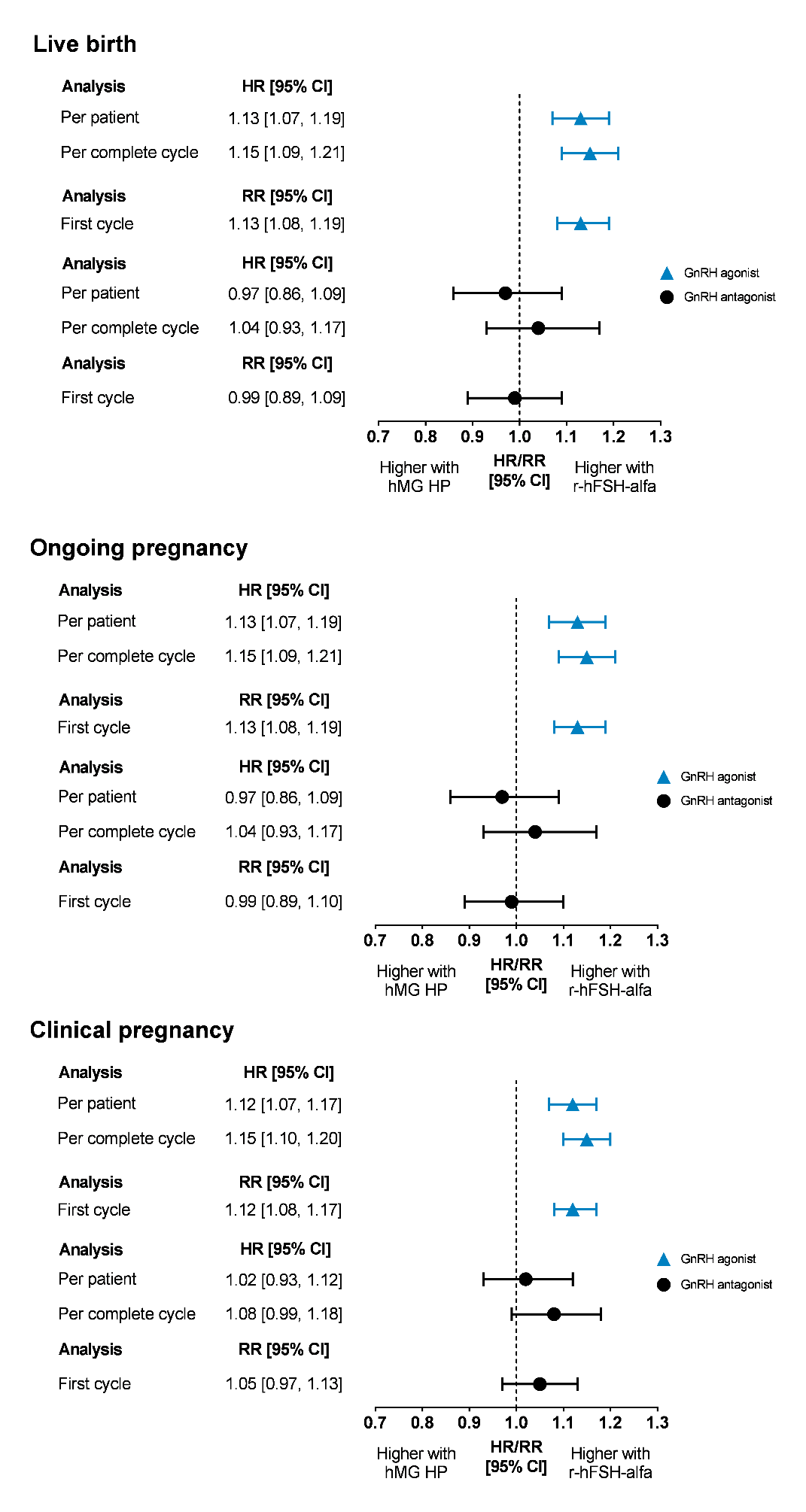


# Supplementary Figure 3


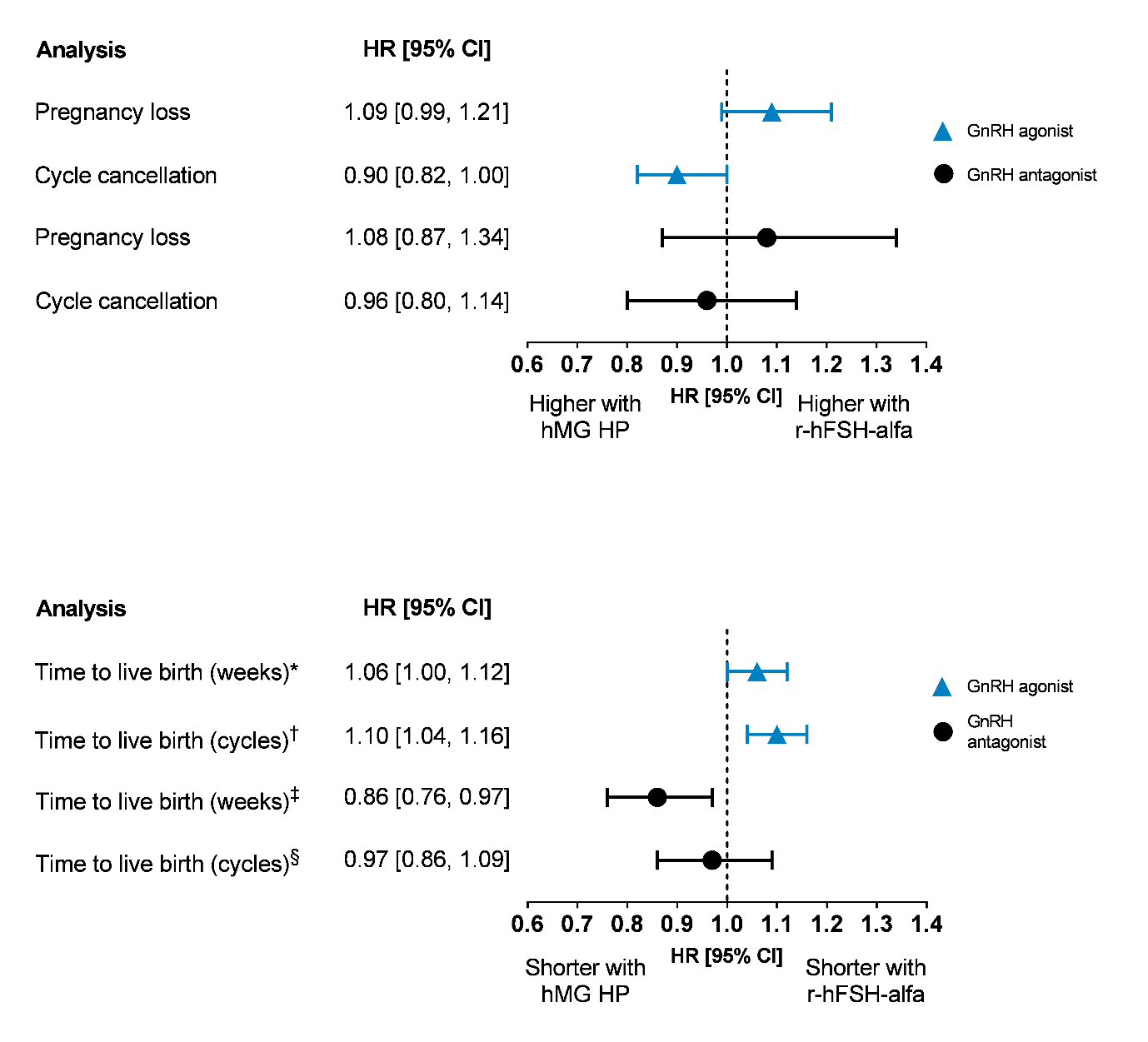

Supplement: Supplementary file 1 — Additional file 1: Supplementary Figure 1. Study design. Supplementary Figure 2. Primary outcomes stratified by GnRH protocol adjusted for possible confounding factors. Supplementary Figure 3. Secondary outcomes stratified by GnRH protocol adjusted for possible confounding factors. [file 12958_2021_768_MOESM1_ESM.docx]
